# Nutrient-Limited Enrichments of Nitrifiers From Soil Yield Consortia of *Nitrosocosmicus*-Affiliated AOA and *Nitrospira*-Affiliated NOB

**DOI:** 10.3389/fmicb.2021.671480

**Published:** 2021-07-12

**Authors:** Jonathan Rodriguez, Seemanti Chakrabarti, Eunkyung Choi, Nisreen Shehadeh, Samantha Sierra-Martinez, Jun Zhao, Willm Martens-Habbena

**Affiliations:** Fort Lauderdale Research and Education Center, Department of Microbiology and Cell Science, University of Florida, Davie, FL, United States

**Keywords:** nitrification, competition, nutrient limitation, ammonia-oxidizing bacteria, ammonia-oxidizing archaea, comammox, complete ammonia-oxidizing bacteria

## Abstract

The discovery of ammonia-oxidizing archaea (AOA) and complete ammonia-oxidizing (comammox) bacteria widespread in terrestrial ecosystems indicates an important role of these organisms in terrestrial nitrification. Recent evidence indicated a higher ammonia affinity of comammox bacteria than of terrestrial AOA and ammonia-oxidizing bacteria (AOB), suggesting that comammox bacteria could potentially represent the most low-nutrient adapted nitrifiers in terrestrial systems. We hypothesized that a nutrient-limited enrichment strategy could exploit the differences in cellular kinetic properties and yield enrichments dominated by high affinity and high yield comammox bacteria. Using soil with a mixed community of AOA, AOB, and comammox *Nitrospira*, we compared performance of nutrient-limited chemostat enrichment with or without batch culture pre-enrichment in two different growth media without inhibitors or antibiotics. Monitoring of microbial community composition via 16S rRNA and *amoA* gene sequencing showed that batch enrichments were dominated by AOB, accompanied by low numbers of AOA and comammox *Nitrospira*. In contrast, nutrient-limited enrichment directly from soil, and nutrient-limited sub-cultivation of batch enrichments consistently yielded high enrichments of *Nitrosocosmicus*-affiliated AOA associated with multiple canonical nitrite-oxidizing *Nitrospira* strains, whereas AOB numbers dropped below 0.1% and comammox *Nitrospira* were lost completely. Our results reveal competitiveness of *Nitrosocosmicus* sp. under nutrient limitation, and a likely more complex or demanding ecological niche of soil comammox *Nitrospira* than simulated in our nutrient-limited chemostat experiments.

## Introduction

Nitrification, the microbial oxidation of ammonia via nitrite to nitrate, is a central process of the global nitrogen cycle and carried out by an increasingly complex network of bacteria and archaea. Ammonia oxidation, the first and often rate-limiting step of nitrification, is now known to be carried out by three distinct lithoautotrophic microbial groups including ammonia-oxidizing archaea (AOA) within the archaeal phylum *Thaumarchaeota*, ammonia-oxidizing bacteria (AOB) within the beta- and gamma-subgroup of *Proteobacteria*, and complete ammonia-oxidizing (comammox) bacteria within the bacterial phylum *Nitrospirae* (Daims et al., 2016; Lawson and Lücker, 2018; Norton and Ouyang, 2019; Prosser et al., 2020). Furthermore, several heterotrophic proteobacterial and fungal taxa have been shown to oxidize ammonia (Prosser, 1989; Stein, 2011). Following more than a century of research primarily focusing on the then only known AOB, the discovery of AOA ubiquitous in marine and terrestrial environments, and comammox bacteria widespread in terrestrial ecosystems have vastly expanded the diversity of autotrophic ammonia oxidizers. These discoveries further raised new fundamental questions about the biology and ecology of ammonia oxidation, the distinct physiology, niche preferences, and specific activities of each group and the associated N_2_O emissions (Könneke et al., 2005; Prosser and Nicol, 2008, 2012; Tourna et al., 2011; Daims et al., 2015; van Kessel et al., 2015; Kozlowski et al., 2016; Jung et al., 2019; Kits et al., 2019).

Important insights into distinct biological traits of AOA, AOB, and comammox nitrifiers have come from studies on genomes and metagenomes of available isolates, enrichments, and natural ecosystems enriched in ammonia oxidizers (e.g., Treusch et al., 2005; Walker et al., 2010; Bartossek et al., 2012; Stahl and de la Torre, 2012; Daims et al., 2015; Santoro et al., 2015, 2017; van Kessel et al., 2015; Kerou et al., 2016; Palomo et al., 2016, 2018; Sauder et al., 2017; Lawson and Lücker, 2018; Stein, 2019; Spasov et al., 2020). However, understanding of the genetic and physiological diversity of nitrifiers in complex systems such as soils and sediments is still limited due to the challenges associated with obtaining high quality draft genomes or genomic inventories of nitrifiers (e.g., Orellana et al., 2018; Kerou et al., 2021). Furthermore, many important biological traits of ammonia oxidizers, such as kinetic properties, adaptation and response to changing environmental conditions (e.g., pH, temperature, oxygen, organic matter), and maybe most significantly their metabolism of nitric oxide (NO) and nitrous oxide (N_2_O), cannot be deducted from genomic sequences alone (Walker et al., 2010; Stahl and de la Torre, 2012; Martens-Habbena et al., 2015; Kozlowski et al., 2016; Lehtovirta-Morley, 2018). Hence, there remains a need for relevant model organisms and integrated physiological studies to inform these complex biological traits and improve interpretation of genetic inventories of nitrifiers (Stahl and de la Torre, 2012; Lehtovirta-Morley, 2018; Stein, 2019; Prosser et al., 2020).

Existing cultivation techniques used to enrich, isolate and study ammonia oxidizers yielded AOB, likely for a combination of reasons including ammonia toxicity, unmatched trace metal requirements or toxicity, pH and temperature adaptation, symbiotic dependencies, such as vitamin and antioxidant requirements (Bollmann et al., 2011; Qin et al., 2014). The recent discoveries of novel nitrifying organisms have benefited from more detailed knowledge of organismal inventories based on molecular studies and also relied on innovative approaches to enrich nitrifiers. For example, the isolation of the first ammonia-oxidizing archaeon, *Nitrosopumilus maritimus* SCM1, came from a marine aquarium devoid of known AOB, spurring the search for novel ammonia oxidizers and enabling systematic variation of cultivation conditions for optimization of ammonia oxidation in the absence of AOB (Könneke et al., 2005; Stahl and de la Torre, 2012; Stahl, 2020). These efforts resulted in identification of the archaeon as the causative agent, and subsequent isolation of strain SCM1 after treatment of the enrichment with bacterial antibiotics (Könneke et al., 2005). Enrichment and isolation of other AOA strains directly from coastal and open ocean seawater and soil also required innovative techniques such as pre-enrichment in original sample water, addition of antioxidants, and application of various antibiotics for enrichment of AOA (e.g., Santoro and Casciotti, 2011; Tourna et al., 2011; French et al., 2012; Qin et al., 2014; Jung et al., 2016). Similarly, the enrichment and isolation of *Ca.* Nitrospira inopinata from the biofilm of a water production pipe of an abandoned oil exploration well at 56°C, was above the growth range of known canonical AOB, facilitating enrichment of the novel organism (Daims et al., 2015). Similarly, *Ca.* Nitrospira nitrosa and *Ca.* Nitrospira nitrificans, two comammox *Nitrospira* species, were successfully enriched along with anammox bacteria from the anaerobic compartment of a trickling filter in a recirculation aquaculture system at low oxygen (3.1 μM), and the enrichment was shown to be devoid of canonical AOB or AOA (van Kessel et al., 2015). However, a vast diversity of ammonia oxidizers inhabit environments where different groups of ammonia oxidizers compete or coexist (e.g., soils, sediments, lakes). Strains representative of the predominant lineages and ecotypes from such environments are still scarce, but are vital to study the adaptations of nitrifiers to these environments and to the competition and coexistence of nitrifiers within the complex ecosystems (Lehtovirta-Morley, 2018; Stein, 2019). Thus, further efforts are needed to improve cultivation success of representative strains.

Kinetic studies on several AOA and *Ca.* N. inopinata indicate that many AOA and comammox bacteria exhibit low apparent half saturation constants [*K*_*m(app*)_] for ammonia (Martens-Habbena et al., 2009; Jung et al., 2011; Kits et al., 2017; Straka et al., 2019; Sakoula et al., 2020). Some strains additionally exhibit high specific substrate affinities and thus appear well-adapted to low substrate concentrations (Martens-Habbena et al., 2009; Jung et al., 2011; Kits et al., 2017; Sakoula et al., 2020). However, Kits et al. (2017) found that *Nitrososphaera*-related AOA and AOB from soil may possess rather similar kinetic properties. This raises the question whether kinetic properties are not a significant selective factor for nitrifiers in soils, or if the ammonia concentrations used during the original enrichments may have selected for less oligotrophic and more ammonia tolerant strains. We therefore aimed to test the hypothesis that a nutrient-limited enrichment strategy could yield novel oligotrophic ammonia oxidizers from terrestrial environments harboring mixed assemblages of AOA, AOB, and comammox bacteria. Using soil samples from highly active agricultural soils with a mixed assemblage of nitrifiers we highly enriched a new AOA strain in nutrient-limited continuous culture setups and a new AOB strain in batch cultures. However, only a weak enrichment of a comammox strain predominant in the soil was obtained in batch culture enrichments.

## Materials and Methods

### Soil Physicochemical Analyses

Soil samples for enrichment of nitrifiers were collected in August 2017 and April 2018 from a histosol soil in the Everglades Agricultural Area in South Florida. In a previous study we found that this soil harbors an AOA-dominated community of nitrifiers that also contains AOB and comammox bacteria (Huang et al., 2021). Concentrations of NH_4_^+^-N and NO_3_^–^-N were determined colorimetrically after extraction with 2M KCl. Briefly, 5 g wet soil was extracted with 45 ml of 2 M KCl solution by shaking for 1 h at 50 rpm. Samples were centrifuged for 10 min at 3,000 *g* and 10 ml supernatant was filtered through a 0.45 μm Nylon filter and refrigerated until analysis within 1–5 days. Ammonium, nitrite and nitrate were determined colorimetrically as described below. Soil pH was determined in a slurry of 1:2 (*w/v*) ratio of soil and deionized water.

### Batch Culture Enrichments

After return to the laboratory in August 2017 batch enrichment cultures were established by adding 0.5 g of field-moist soil to 50 ml of synthetic freshwater media (SFCM) in 250 ml Pyrex glass bottles. The bottles were tightly closed and incubated at 28°C in the dark without shaking. The SFCM media contained (per 1 l) 1.0 g NaCl, 0.4 g MgCl_2_.6 H_2_O, 0.1 g CaCl_2_.2 H_2_O and 0.5 g KCl (de la Torre et al., 2008). After autoclaving the media was cooled to room temperature and supplemented with the following sterile stock solutions (per 1.0 l): 2.0 ml NaHCO_3_ (84 g l^–1^), 5.0 ml KH_2_PO_4_ (0.4 g l^–1^), 1.0 ml FeNaEDTA (2.75 g l^–1^), 1.0 ml modified non-chelated trace element solution (Martens-Habbena et al., 2009). If not otherwise mentioned the medium was supplemented with 0.5 ml of NH_4_Cl (1.0 M) per 1.0 l medium. The pH of this medium at room temperature was ∼7.5.

Enrichment cultures were monitored for ammonia consumption and nitrite production and transferred to fresh media with 1% inoculum once 70–80% of the ammonia was consumed. Following twelve consecutive transfers over ∼ 6-month period the ammonium concentration was reduced to 300 μM, and following another 6 months of consecutive 1% transfers (equivalent to a 10^58^ final dilution of the original soil sample), one culture (batch enrichment culture 9) was selected and scaled up from 50 to 400 ml volume sufficient for DNA extraction (see below).

### Bioreactor Enrichments

**FIGURE 1:**
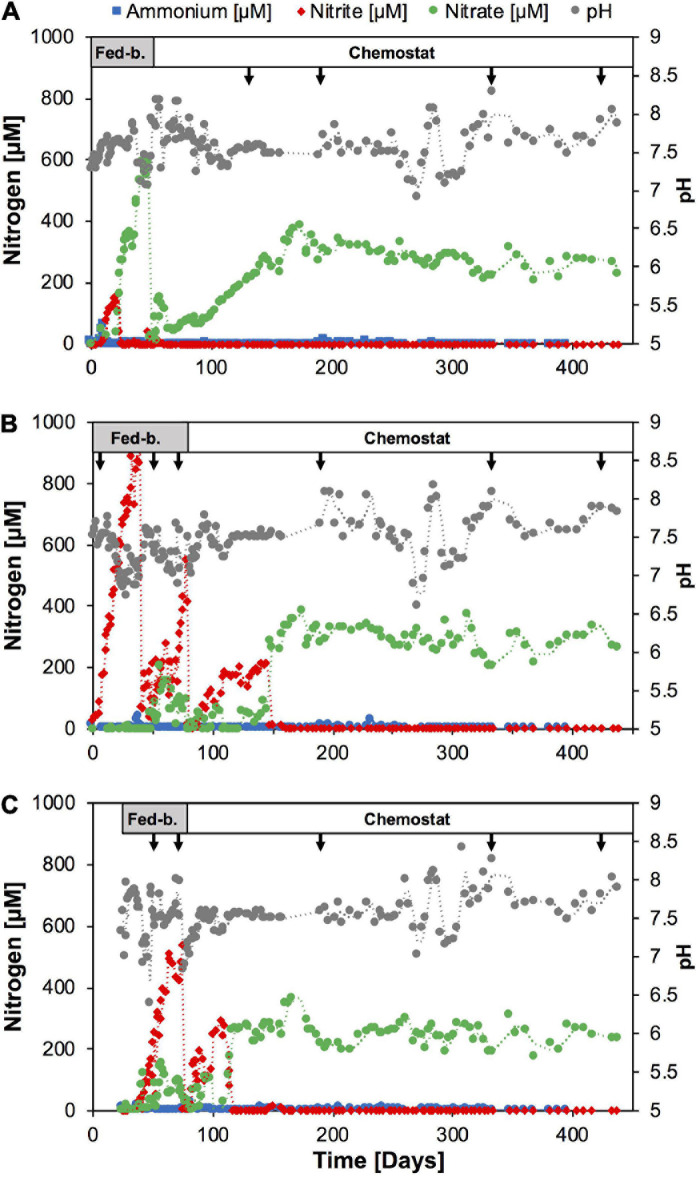
Time course of nutrients and pH during continuous culture enrichments of nitrifiers directly from soil with SFCM media in reactor A **(A)**, and following batch culture pre-enrichment, in SFCM media in reactor B **(B)** and modified NGM media in reactor C **(C)**, respectively. Arrows indicate sampling points for amplicon sequencing analysis. Reactor operation modes are indicated on top. Fed-b., Fed-batch mode; Chemostat, chemostat mode. See Supplementary Figure 1 for nutrient and pH time course in subculture chemostat reactors.

In order to test the effect of nutrient limitation on the enrichment of nitrifiers, we established three continuous cultures in 3-l bioreactors (New Brunswick Scientific Bioflo 110) with 1.0 l working volume. Reactor A (Figure 1A) was established directly from the April 2018 soil sample, and reactors B and C were established from the scaled-up batch enrichment culture 9 described above. The soil sample (4.0 g) was mixed with 40 ml of SFCM media (see above), vortexed vigorously for 3 min to remove cells from soil particles and centrifuged at 300 *g* to remove coarse soil particles. A 5 ml volume of the soil-free sample was then directly introduced into the bioreactor vessel with 1.0 l working volume of ammonium-free SFCM media (see above) buffered with 20 mM NaHCO_3_. The reactor feed was composed of the same media amended with 1 mM NH_4_Cl (fed-batch period) and subsequently reduced to 0.3 mM (chemostat mode). The reactor was temperature-controlled at 28°C and stirred at 50 rpm. For pH balance and to avoid atmospheric contaminations the reactor was operated under synthetic air headspace (5% CO_2_, 95% synthetic air). It was operated in fed-batch mode for the first approximately 40 days. During the first 2 weeks after start-up, the feeding rate was slowly raised from ∼1.5 ml per day to 30 ml per day and maintained at 30 ml per day for approximately 25 days. Once the volume exceeded 2.0 l culture liquid was pumped out to reduce the working volume back to 1.0 l. At a NO_3_^–^ concentration of ∼600 μM the reactor was switched to chemostat mode and operated continuously for the following 400 days with a 0.3 mM NH_4_Cl feed concentration. From Day 41 to Day 120 the feeding rate was slowly raised from 30.0 ml per day (33 days hydraulic retention time) to 200 ml per day (5 days hydraulic time) and remained constant thereafter.

Reactors B and C (Figures 1B,C) were established from 5 ml inoculum of batch enrichment culture (see above) in 1.0 l ammonium-free SFCM media and 1.0 l ammonium-free modified NGM media (Sauder et al., 2017), respectively. The modified NGM media was prepared as follows: 50 ml of a 20x basal salt solution (11.68 g NaCl, 3.0 g CaCl_2_.2H_2_O, 1.5 g KCl, 1.0 g MgSO_4_.7H_2_O per 1.0 l) were added to 930 ml MilliQ water, autoclaved, and cooled to room temperature. The media was supplemented with the following sterile stock solutions (per 1.0 l): 20 ml NaHCO_3_ (84 g l^1^), 5.0 ml KH_2_PO_4_ (0.4 g l^–1^), 1.0 ml FeNaEDTA (2.75 g l^–1^), 1.0 ml trace element solution (Daims et al., 2015). The final pH of this medium at room temperature was ∼7.5. The feed media for both reactors was initially supplemented with 1.0 mM NH_4_Cl (fed-batch period) and 0.3 mM NH_4_Cl (chemostat period) and both reactors were operated at 28°C with 50 rpm stirring and synthetic air headspace (5% CO_2_, 95% synthetic air). Both reactors were operated in fed-batch mode and the feeding rate was raised from 1.5 ml per day to approximately 50 ml per day and 30 ml per day for reactors B and C, respectively. Similar to reactor A, liquid was pumped out to reduce the working volume back to 1.0 l once the volume exceeded 2.0 l in reactors B and C, respectively. The reactors were switched to chemostat mode at Day 78 and Day 74, respectively. Subsequently, both reactors B and C were operated in chemostat mode continuously for the following ∼380 days. The feeding rate was slowly raised to 200 ml per day (5 days hydraulic retention time) until Day 173 and Day 131 in reactor B and C, respectively, and remained constant thereafter.

To test whether higher enrichments could be obtained through either more frequent transfers to fresh reactors, increased NH_4_Cl feed concentration, or reduction of organic carbon through removal of EDTA from the Fe-trace metal solution, three consecutive nutrient-limited subculture reactors were run with shorter operation times using the same general operation conditions and feeding rate of 200 ml per day (5-day hydraulic retention time). The first subculture reactors (subculture 1) were set up with 100 ml culture liquid from reactor A, B, and C, respectively. In subculture reactors A and B the feed NH_4_Cl concentration was raised to 1.0 mM after 48 days of operation. After approximately 80 days, 800 ml inoculum of subculture 1 were transferred to subculture 2, again operated for approximately 80 days, and subsequently 100 ml inoculum of subculture 2 was used to establish subculture 3. For subcultures 3, the NH_4_Cl feed concentration was further raised to 1.8 mM and FeEDTA was replaced with FeSO_4_ as iron source at the same concentration. From reactor C only one subculture reactor was set up and run for 120 days and the ammonium feed concentration was maintained at 0.3 mM. Samples for DNA isolation and amplicon sequencing were taken before each sub-cultivation.

Samples for nutrient analyses were collected 2–3 times per week (reactors A, B, C) or 1–2 times per week (subculture reactors) and analyzed as described below. Samples for microbial community analysis were collected at time points indicated in Figure 1 and Supplementary Figure 1. Before sampling for DNA sequencing, reactor outflow was stopped to allow accumulation of culture volume to approximately 2 l, with the exception of reactor B at Day 7, when just 200 ml culture sample was taken from the reactor. Glass surfaces were scraped with a silicon spatula to loosen biofilm if present. Then 1-l samples from each reactor were collected in Pyrex bottles for DNA isolation and cells were harvested by filtration onto Sterivex filters (Urakawa et al., 2010). DNA was isolated using the procedure described by Griffiths et al. (2000) with modifications described by Nicol et al. (Nicol and Prosser, 2011).

In order to isolate the predominant AOA strain, duplicate batch cultures were set up with 1–10% inoculum from reactor A or subculture reactor 1A in 250-ml Pyrex glass bottles using 50 ml SFCM media (as described above) with either 50 μM or 1.0 mM NH_4_Cl and either of the following antibiotics: streptomycin (50 μg/ml final concentration), kanamycin (50 μg/ml), ampicillin (50 μg/ml), ciprofloxacin (10 μg/ml), azithromycin (10 μg/ml), lincomycin (50 μg/ml), as well as duplicate uninhibited controls. Growth was assessed over a 4-week period by ammonium consumption (50 μM NH_4_Cl cultures) or nitrite and nitrate formation (1.0 mM NH_4_Cl cultures) relative to uninhibited controls. In additional experiments, the antibiotic treatments were combined with supplementation of either sodium pyruvate, α-ketoglutarate, or malic acid (100 μM final concentration).

### Analytical Methods

Ammonium and nitrite concentrations in batch and bioreactor samples and KCl extracts of soil samples were determined using the salicylate-trichloroisocyanuric acid method (Bower and Holm-Hansen, 1980) and sulfanilamide-NED method (Grasshoff et al., 1999), respectively. For accurate determination of ammonium in the bioreactor samples, the pH of the alkaline trichloroisocyanuric acid reagent was adjusted to compensate for the 20 mM bicarbonate buffer in the reactor media without having to dilute the reactor samples. Nitrate concentrations were determined using the method by García-Robledo et al. (2014). Briefly, 150 μL VCl_3_ reagent (2% w/v VCl_3_ in 6.0 M HCl) and 1.0 ml sample were combined in 1.5 ml reaction tubes, mixed, and incubated at 60°C for exactly 100 min. Combined NO_2_^–^ and NO_3_^–^ were subsequently determined colorimetrically using the modified sulfanilamide-NED reagent as described (García-Robledo et al., 2014).

### *amoA* Gene Sequence Analyses

Gene fragments of target *amoA* were PCR-amplified using previously described primers for archaeal *amoA* (Tourna et al., 2008), bacterial *amoA* (Rotthauwe et al., 1997), and comammox *amoA* (Pjevac et al., 2017). PCR reactions (25 μl) contained 12.5 μl of GoTaq PCR Master Mix (Promega, Fitchburg, WI, United States), 1 μl each of forward and reverse primer (0.5 μM final concentration for archaeal and bacterial *amoA*, 0.25 μM final concentration of each forward primer comaA-244f_a-f and reverse primer comaA-659r_a-f for comammox *amoA*) and 2 μl of DNA template (∼5 ng/μl). PCR cycling conditions were as follows: Initial denaturation at 94°C for 5 min, followed by 30 cycles of denaturation at 94°C for 30 s, annealing at 52°C (53°C for comammox *amoA*) for 30 s, and primer extension at 72°C for 45 s, followed by 10 min final extension at 72°C.

PCR products were purified using Qiagen PCR purification kit (Qiagen, MD, United States) and sequenced by Sanger sequencing at Eurofins Genomics (Huntsville, AL, United States). Sequences were manually quality-checked and imported into ARB (Ludwig et al., 2004). For analysis of comammox *amoA* sequences an ARB-formatted alignment provided by Pjevac et al. (2017) was used and amended with additional sequences from GenBank and the present study. Sequences were aligned and maximum likelihood phylogenetic trees were calculated based on 595, 453, and 383 nucleotide positions for archaeal, bacterial, and comammox *amoA*, respectively, using the RAxML program with GTRGAMMA-25 rate distribution model and rapid hill climbing algorithm.

### High-Throughput Amplicon Sequencing of 16S rRNA Genes

The workflow for 16S rRNA gene amplicon sequence analysis followed Earth Microbiome Project standard protocols (Thompson et al., 2017). Briefly, the V4 region of the 16S rRNA gene was amplified using primers 515F (Parada et al., 2016) and 926R (Quince et al., 2011). The forward primer included sequencing adapter sequences and the reverse primer contained the twelve base barcode sequence. PCR reactions consisted of 9.5 μl DNA-Free PCR Water (MoBio, Carlsbad, CA, United States), 12.5 μl 2x AccuStart II PCR ToughMix (Quantabio, Beverly, MA, United States), 1.0 μl 200 pM forward primer, 200 pM Golay barcode-tagged reverse primer, and 1.0 μl template DNA. All PCR template DNA was normalized to ∼20 ng/μl. PCR cycling conditions were: denaturation at 94°C for 3 min, 35 cycles at 94°C for 45 s, 50°C for 60 s, and 72°C for 90 s; and a final extension step of 10 min at 72°C. PCR products were quantified using PicoGreen (Invitrogen, Carlsbad, CA, United States) in a 96 well microplate reader (Infinite 200 PRO, Tecan, Grödig, Austria) and pooled in equimolar amounts, purified using AMPure XP Beads (Beckman Coulter, Brea, CA, United States), quantified by Qubit DNA quantification kit (Invitrogen, Carlsbad, CA, United States), diluted to 2 nM and denatured. Samples were then diluted to final concentration of 6.75 pM with a 10% PhiX spike. The libraries were sequenced on a Illumina MiSeq instrument or a Illumina NovaSeq instrument. PCR amplifications, library preparations and MiSeq DNA sequencing were conducted either at the Environmental Sample Preparation and Sequencing Facility (ESPSF) at Argonne National Laboratory or Novogene, Sacramento, CA, United States.

Amplicon sequences were demultiplexed on the instrument. The command line interface of the QIIME 2 package was used for downstream analyses (Bolyen et al., 2019). DADA2 (Callahan et al., 2016) was used with default settings for quality trimming, denoising and chimera removal and to generate amplicon sequence variants (ASVs). On average 32,164 high quality sequences were obtained per sample, with exception of reactor B (Day 89) and reactor C subculture 1 that had the lowest coverages with 1,822 and 2,553 high quality sequences, respectively. After removing ASVs with less than 4 sequences in at least one sample a total of 2,724 ASVs were identified among all samples. Taxonomic assignments were added to ASVs using the Qiime feature-classifier with sklearn algorithm against the Silva database version 132 (Quast et al., 2012). Sequences of mitochondria and chloroplasts were removed. Sequences of ASVs affiliated with known genera of archaeal and bacterial nitrifiers were imported into ARB and manually aligned. Backbone phylogenetic trees were calculated with near full-length 16S rRNA gene sequences using the accelerated maximum-likelihood method with positional variability filters for *Archaea*, *Betaproteobacteria*, and *Nitrospira*, respectively, and ASV sequences were inserted using the “add sequences using parsimony” option in ARB with the same filters and *Escherichia coli* position limits 534 and 906. Diversity metrics including Shannon Index and Observed_OTUs were calculated using the “qiime diversity core-metrics-phylogenetic” function in Qiime 2 after rarefaction to 1,200 sequences.

## Results

In this study we employed two complementary strategies for enrichment of nitrifiers at low substrate concentrations from a highly fertile, unfertilized agricultural soil. We previously found this soil to contain between 1 × 10^8^ and 5 × 10^8^ archaeal *amoA* genes g^–1^ soil and between 1 × 10^6^ and 5 × 10^6^ bacterial and comammox *amoA* genes g^–1^ soil, respectively (Huang et al., 2021). The physicochemical properties of the soil samples were as follows: The pH was 7.3, the concentrations of NH_4_^+^-N ranged between 5.6 and 8.6 μg N g^–1^ dry soil and NO_3_^–^-N ranged between 43.8 and 116.1 μg N g^–1^ dry soil, respectively.

**FIGURE 2:**
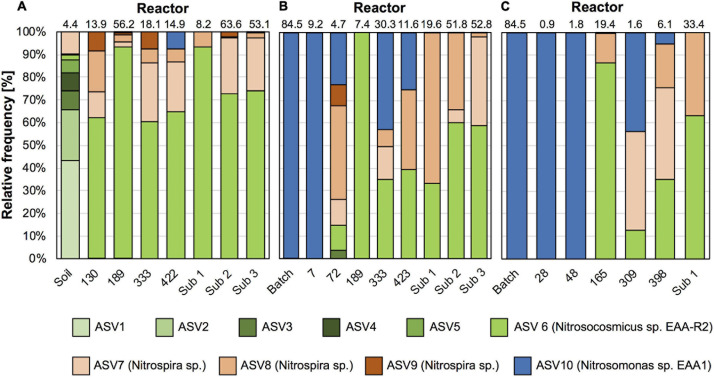
Time series of nitrifier community composition in reactors **A**, **B**, and **C**. Given are relative frequency of 16S rRNA genes affiliated with thaumarchaeal AOA (green), betaproteobacterial AOB (blue), and *Nitrospira* sp. (beige) of all nitrifier-associated sequences. Numbers on top indicate relative abundance of nitrifiers in each whole community sample in%. Sub 1, Sub 2, and Sub 3 refer to sub-culture reactors 1, 2 and 3, respectively.

**FIGURE 3:**
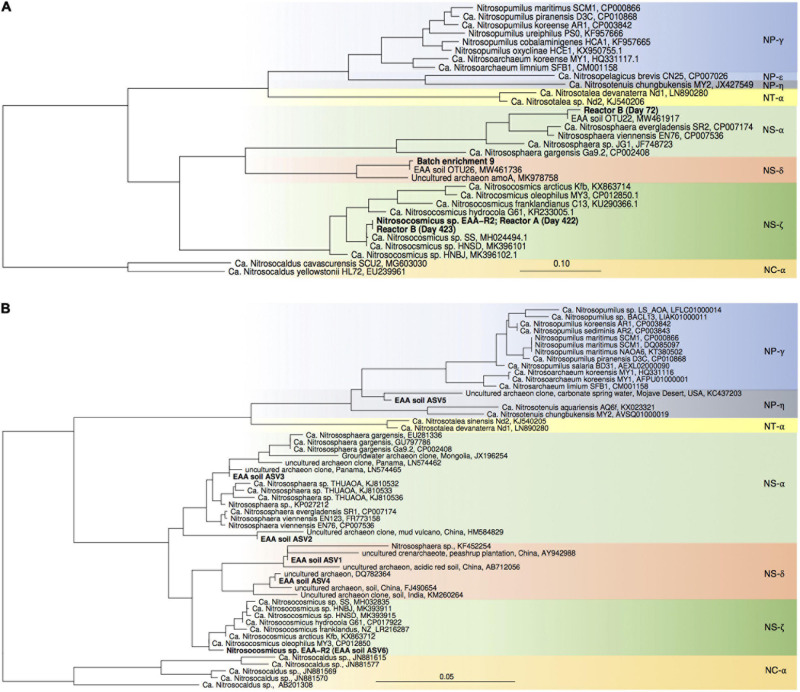
Maximum-likelihood phylogenetic trees of archaeal *amoA* gene sequences **(A)** and thaumarchaeal 16S rRNA gene sequences **(B)**. Sequences obtained in this study are highlighted in bold. *Nitrosocaldus* sp.-associated sequences were used as outgroup. Color codes refer to archaeal lineages (after Alves et al., 2018).

High throughput sequencing of 16S rRNA gene from April 2018 confirmed our previous results, showing that AOA dominated over bacterial nitrifiers in this soil. In total 4.4% of all 16S rRNA gene sequences belonged to nitrifiers (i.e., AOA, AOB, and *Nitrospira*), of which 90.1% were affiliated with AOA and 9.9% with *Nitrospira*. Similar to our previous study, AOB-affiliated 16S rRNA gene sequences were not detected. AOA-affiliated sequences were dominated by 6 ASVs with proportion of 0.1–2.1%, and the ASV representing the most abundant AOA were assigned to an uncultivated *Nitrososphaera* sister group (NS-δ; Figures 2, 3).

**FIGURE 4:**
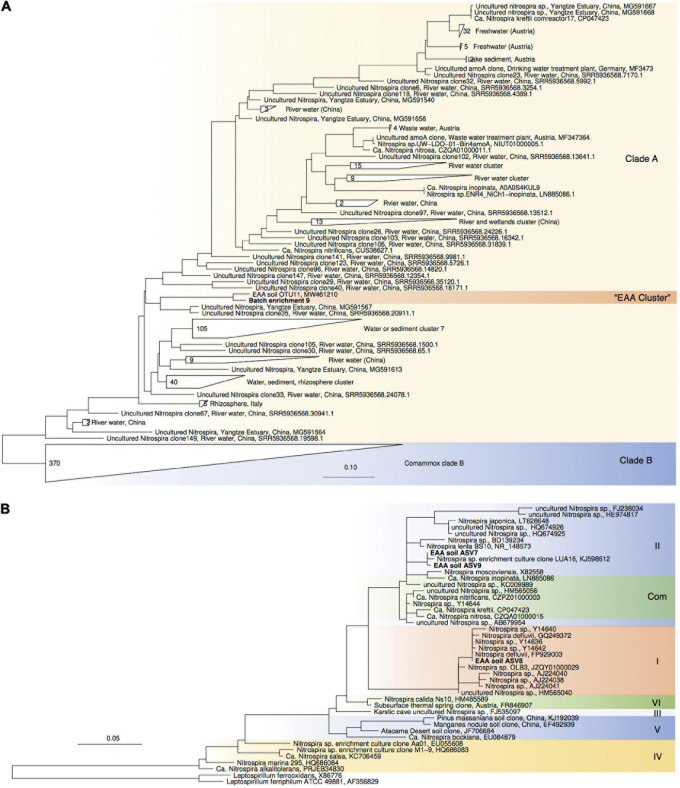
Maximum-likelihood phylogenetic trees of comammox *amoA* gene sequences **(A)** and *Nitrospira* sp.-affiliated 16S rRNA gene sequences **(B)**. Sequences obtained from batch enrichment culture 9 are highlighted in bold. Comammox clade B sequences, and *Leptospirillum* sp. sequences were used as outgroups, respectively.

**FIGURE 5:**
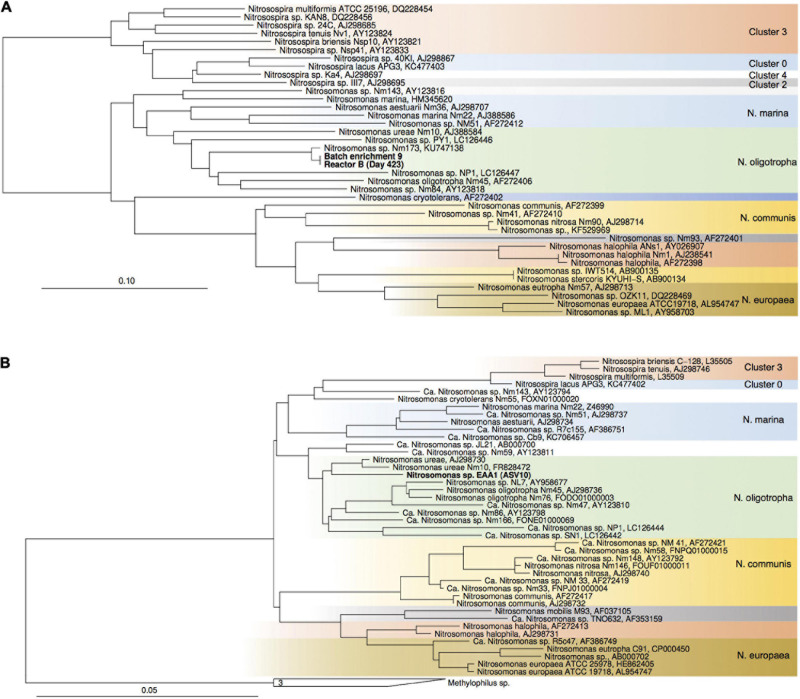
Maximum-likelihood phylogenetic tree of proteobacterial *amoA* gene sequences **(A)** and 16S rRNA genes of *Nitrosomonadaceae*-affiliated AOB **(B)**. Sequences from the current study are highlighted in bold. *Nitrosococcus*- and *Methylophilaceae*-affiliated sequences were used as outgroups, respectively. Color codes highlight *Nitrosospira sp.* clusters and *Nitrosomonas* lineages.

For enrichments we used a basal media composition conducive to cultivation of AOB and AOA (Martens-Habbena et al., 2015). Batch enrichments were carried out initially at 500 μM NH_4_Cl, sufficient to allow for growth of AOA, AOB and comammox based on available kinetic data. Out of 10 different enrichments monitored over a 6-month period, 9 enrichments yielded stable nitrite production within 1–2 month. Following repeated approximately bi-weekly transfers over a 6-month period, nitrite accumulation and microscopic analysis suggested similar composition and nitrifying activities in most of the batch cultures. Therefore, one culture (batch enrichment 9) was selected and characterized in detail after additional twelve consecutive 1% transfers over ∼ 6-month period (equivalent to a 10^–58^ final dilution of the original soil sample). PCR analysis revealed presence of archaeal, bacterial and comammox *amoA* genes and sequencing of purified PCR products revealed unique and unambiguous *amoA* gene sequences for all three groups (Figures 3A, 4A, 5A), suggesting dominance of a single *amoA* type of each group in the enrichment. Intriguingly, the AOA *amoA* gene sequence in the batch enrichment was closely related to an AOA phylotype (EAA soil OTU26) within the uncultivated soil *Nitrososphaera*-sister group (NS-δ), previously shown to be frequent in this soil (Figure 3A; Huang et al., 2021). The AOB *amoA* sequence in the batch enrichment affiliated with the *Nitrosomonas* sp. Nm173 lineage which we did not previously observe in this soil, and which has not been described in detail. The comammox *amoA* sequence was affiliated with the most frequent comammox *amoA* sequence type previously found in the soil (EAA soil OTU11, Figure 4A). Together, these data suggested that environmentally relevant AOA and comammox *Nitrospira* strains were maintained in this culture for more than 1 year. Analysis of 16S rRNA gene amplicons showed that the batch enrichment was highly dominated by the *Nitrosomonas* Nm173-affiliated strain (84.5%) and neither archaeal or comammox ammonia oxidizers, or canonical nitrite oxidizers were detectable in the amplicon dataset (Figure 2).

Therefore, we tested in 1.0-l scale bioreactors whether nutrient limited growth conditions would bias the enrichment toward comammox or AOA, or whether the *Nitrosomonas* strain would indeed continue to outcompete other ammonia oxidizers. We seeded one reactor directly with soil biomass (reactor A, Figures 1A, 2A) and two reactors with 5.0 ml inoculum of the batch enrichment 9 using either SFCM medium (reactor B), or a modified NGM medium, similar to that used previously for the isolation of *Ca.* N. inopinata (reactor C). All reactors were initially operated in fed-batch mode and then switched to chemostat mode after stable nitrite or nitrate production was observed (Figure 1). Ammonium concentrations remained below 1 μM in all three reactors for the entire operation time with only few exceptions, indicating high specific ammonia oxidation activities at sub-micromolar substrate concentrations (Figure 1). In reactor A nitrite initially accumulated for approximately 20 days, before nitrate became the sole detected nitrification product. Reactors B and C seeded with enrichment culture biomass rapidly produced nitrite over the first 20–40 days. Intriguingly, both reactors B and C also began to produce nitrate at day 48 and day 30, respectively, although no nitrate production had been observed in the batch enrichment culture itself. In order to test whether higher ammonium feed concentrations and more frequent sub-cultivation could yield higher relative enrichment of nitrifiers, reactors A and B were sequentially sub-cultivated three times (subculture 1–3, Supplementary Figure 1) and ammonium feed concentration was first raised to 1.0 mM (subculture 1) and later to 1.8 mM (subculture 3). Additionally, reactor C was sub-cultured once at the original ammonium feed level. In each of the subculture reactors ammonium was detectable only directly after seeding the reactors, and subsequently fell below 1 μM. Nitrite was only detected at the beginning of subculture B1. As expected, nitrate was the main product and after raising feed concentrations increased as expected to ∼1 mM in subcultures A1 and B2, and ∼1.8 mM in A3 and B3, respectively.

Monitoring of the community composition in all three reactors over approximately 400-day operation, as well as in sub-culture reactors revealed selection for a single *Nitrosocosmicus*-affiliated ASV (ASV 6) in all three reactor sets. Reactor A seeded with microbial biomass directly from soil was dominated by this ASV from the first sampling point at Day 130 until the third sub-culture, accounting for 60.6% and 93.3% of all nitrifier-affiliated sequences (Figures 2, 3B). This AOA type was accompanied by three distinct *Nitrospira* strains (ASV 7–9) with proportions between 0.3 and 25.6% of all nitrifier-affiliated sequences in reactor A. Intriguingly, ASV 7 represented the most frequent *Nitrospira* sp. sequence type found in the soil (Figures 2, 4B), and it remained the dominant *Nitrospira* sequence type in reactor A and subsequent sub-culture reactors.

In reactors B and C, seeded with enrichment culture 9, the *Nitrosomonas* strain (ASV 10) remained predominant during the initial fed-batch operation stage irrespective of growth media used (SFCM vs. NGM in reactor B and C, respectively). However, after onset of chemostat operation, its relative abundance dropped below 0.1% after 189 and 165 days in both reactors B and C, respectively (Figures 2, 5B). Concomitantly, the *Nitrosocosmicus* strain (ASV 6) and *Nitrospira* strains (ASV 7–9) rose in relative abundance from Day 72 and thereafter in reactor B, and Day 165 and thereafter in reactor C. However, *Nitrospira* ASV 9 was absent in reactor C. It remains unclear why the *Nitrospira* sp. (ASV 7–9) were below 0.1% at Day 189 in Reactor B, while steady nitrate production was evident during this period.

Notably, *Nitrosomonas* (ASV 10) became significant again at days 333 and 423 in reactor B, and days 309 and 398 in reactor C, respectively, as well as day 422 of operation of reactor A (Figure 2). In reactor A it declined again below the detection limit, however, in reactor B and C it remained present. Only in the subculture reactors it was not detected. Although we can only speculate about the reasons for this, pH values fluctuated due to interruption of headspace CO_2_ flow over an approximately 2-month period between approximately days 200 and 320 (Figure 1). Following this period pH values were on average 0.19, 0.26, and 0.21 units higher than during the first 200 days of operation in reactors A, B, and C, respectively. These observations show that despite being undetectable by amplicon sequencing in reactor A for most of the time, a small *Nitrosomonas* population was present and survived for more than 400 days of continuous nutrient limitation and successfully competed for nitrogen when conditions became favorable. Its absence in the subculture reactors may suggest that it primarily resided in biofilms on the glass surface of the reactor vessels.

PCR tests for comammox *amoA* remained negative in all reactor DNA samples with the full combination of comaA-244f_a-f and comaA-659r_a-f, as well as the specific primer combination comaA-244f_a and comaA-659r_c targeting the *amoA* sequence identified in the batch enrichment culture 9. It should be noted that the employed comammox *amoA* primers have been shown to have mismatches to some comammox *amoA* sequence types (Lin et al., 2020). However, since the comammox sequence type predominant in our soil was detected with this primer set in batch cultures, our results indicated that the three detected *Nitrospira* ASVs represented canonical nitrite-oxidizing strains and that *Nitrosocosmicus* was the predominant ammonia oxidizer. Further experiments are needed to test additional conditions and improve the enrichment of comammox *Nitrospira*.

In order to investigate competition between different nitrifiers under nutrient-limited conditions, we adopted an enrichment strategy without selective inhibitors. The overall enrichment of nitrifiers in the reactors was notably low. Based on 16S rRNA amplicon sequences, nitrifiers constituted between 0.9 and 56.2% of all 16S rRNA gene reads in reactor sets A–C and never became highly dominant (Figure 2). The highest relative enrichments were observed temporarily at 189 days in reactor A, and in subculture reactors 2 and 3. Increasing the feed ammonium concentration in subculture reactors 1 and 3 led to increased enrichment to above 50%. However, removal of the EDTA solution in subcultures 3 had no instantaneous effect on the community composition or enrichment factor.

**FIGURE 6:**
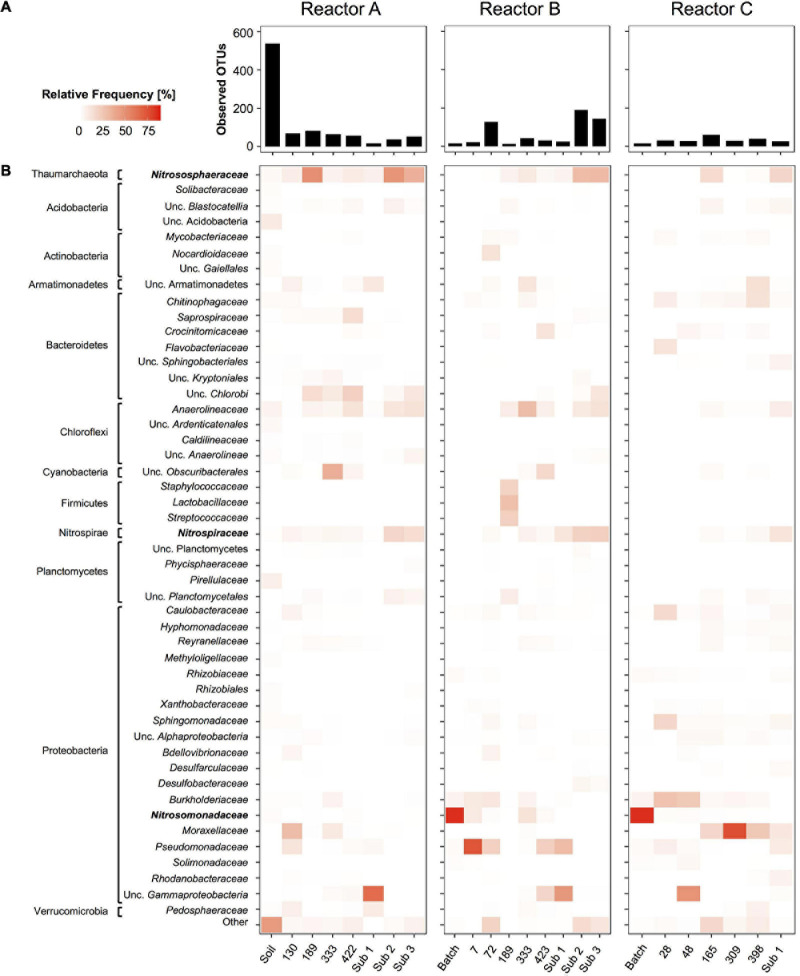
Time series of overall community composition in reactors A, B, and C based on 16S rRNA gene amplicon sequences. Given are overall diversity of bacterial and archaeal taxa as Observed OTU’s at ASV level (bar graphs, **A**) and relative frequency of microbial taxa summed and grouped at the family level (heatmaps, **B**). Family level taxa with more than 1% relative frequency in at least one sample are shown on the left. All other less frequent taxa were grouped into “Other.” For uncultivated taxa without family level taxonomic names next higher level of taxonomic names are given. Nitrifier taxa highlighted in bold are analyzed in more detail in Figure 3. Sub 1, Sub 2, and Sub 3 refer to sub-culture reactors.

Analysis of overall microbial community composition in reactor sets A, B, and C revealed 48 family level archaeal and bacterial taxa with at least 1.0% relative frequency in one of the reactor samples (Figure 6). Overall diversity was high in the soil sample, and as expected dropped quickly in the enrichment cultures (Figure 6A). However, observed OTU values ranging from 18 to 83, 13 to 191, and 16 to 60 in samples from reactor sets A, B, and C, respectively, indicated a surprisingly high microbial diversity persisting throughout the three reactor series.

Each reactor series showed a unique microbial profile. Besides, the nitrifying taxa, only a few bacterial taxa were found consistently throughout each enrichment time series. In reactor set A, uncultivated *Chlorobi*, *Anaerolineaceae*, and *Pseudomonadaceae*-associated sequences were found frequently throughout the time series, whereas in reactor set B, *Chlorobi* were less common and *Anaerolineaceae*, *Burkholderiaceae*, *Pseudomonadaceae*-associated sequences were more often present. In reactor set C, *Burkholderiaceae* and *Moraxellaceae* were the most commonly found non-nitrifying taxa. However, most taxa were rare and only found once or twice with relative frequencies above 1.0% (Figure 6B).

In order to isolate the predominant *Nitrosocosmicus* strain into pure culture, batch cultures were set up with low (50 μM) or high (1.0 mM) ammonium concentration. Several antibiotics were tested that have previously been used successfully to enrich or isolate AOA strains (Lehtovirta-Morley et al., 2016; Sauder et al., 2017; Liu et al., 2019). Within 2 weeks of incubation ammonium was completely consumed in the low ammonium culture controls, and more than 100 μM nitrite/nitrate was produced in the high ammonium culture controls. However, in the kanamycin, ampicillin, ciprofloxacin, azithromycin treatments less than 10 μM ammonia consumption (low ammonium cultures) or combined nitrite and nitrate production (high ammonium cultures) was observed even after more than 4 weeks of incubation. Some ammonia consumption was detected in the streptomycin and lincomycin treatments. However, microscopic examination showed a mixed assemblage of morphotypes in these two treatments, indicating lack of inhibition in these two treatments (data not shown). To further test whether the inhibition of growth in the presence of antibiotics was due to lack of organic carbon source or antioxidants previously shown to be required by some *Nitrosopumilus* and *Nitrososphaera* strains in batch culture (Tourna et al., 2011; Qin et al., 2014; Kim et al., 2016), additional experiments were conducted with combinations of organic carbon compounds (pyruvate, alpha-ketoglutarate, or malic acid) with kanamycin or ampicillin treatment. However, similar to the antibiotic treatments alone, no significant ammonia oxidation activity was observed after more than 1 month incubation (data not shown).

## Discussion

The recognition of the vast diversity of AOA and comammox bacteria adapted to low ammonium fluxes still awaiting cultivation and physiological characterization has renewed interest in improved cultivation methods targeting oligotrophic nitrifiers (Bollmann et al., 2011). Significant improvements have been made in overall cultivation success of microbes by better simulating environmental conditions and decreasing nutrient concentrations (Bollmann and Laanbroek, 2001; Overmann, 2010; Lewis et al., 2021). However, enrichment of nitrifiers still remains extremely difficult (Bollmann et al., 2011). Recent studies indicated that available soil AOA strains exhibit kinetic properties similar to low-nutrient adapted soil AOB isolates, e.g., of the *Nitrosomonas oligotropha* lineage, suggesting that kinetic properties may possibly not be a distinguishing factor between these soil AOA and AOB, and that comammox bacteria may be more adapted to nutrient limited conditions (Kits et al., 2017). In support of these results, recently comammox *Nitrospira* were successfully enriched in nutrient-limited membrane bioreactors with biomass retention (Sakoula et al., 2020), sequencing batch reactors (van Kessel et al., 2015; Camejo et al., 2017), and in reactors with fabrics as biomass carriers (Takahashi et al., 2020). Although in our study the comammox *Nitrospira* strain was detected in batch culture after more than a year of bi-weekly transfers (equivalent to a 10^–58^ dilution of the original soil), clearly indicating growth in the batch cultures, no further enrichment of this strain was obtained in the nutrient-limited chemostat setups employed with either of the tested growth media. The low cell density in the cultures and the limited sample DNA from each sampling point did not allow us to further evaluate changes immediately after transfer to the bioreactors or at higher frequency during reactor operation, or to perform additional qPCR tests to characterize populations below the detection limit of the amplicon sequencing dataset.

The main difference between our chemostat setups and the reactor setups employed in the above studies was that our chemostats did not contain a mechanism of biomass retention. Several comammox strains cultivated thus far originate from environmental biofilms (e.g., *Ca.* N. inopinata, *Ca.* N. nitrificans, *Ca.* N. nitrosa, Daims et al., 2015; van Kessel et al., 2015) and have been shown to form aggregates or biofilms in culture (van Kessel et al., 2015; Sakoula et al., 2020; Takahashi et al., 2020). Metagenomic studies have found comammox to be abundant in biofilms, e.g., of groundwater wells, drinking water treatment systems, and freshwater biofilters (Daims et al., 2015; Pinto et al., 2015; Palomo et al., 2016; Bartelme et al., 2017). Together these findings suggest that comammox bacteria may have adopted a biofilm lifestyle, or at least grow best in biofilms or aggregates, or higher cell densities, and may not be as competitive in classical chemostat culture without biomass retention. Indeed, Costa et al. (2006) predicted based on kinetic theory of optimal pathway length that a comammox organisms should be most competitive under slow, substrate-limited growth in flocs, biofilms, or microcolonies. Biomass retention might be especially important and a strong selection factor at low initial cell densities and therefore may have prevented a successful establishment of comammox in our reactors. These results may also suggest that biofilm lifestyle is also the preferred lifestyle of comammox bacteria from EAA soils.

Several additional factors may be responsible for the lack of further enrichment of comammox strains from EAA soils. These include suboptimal temperature, too high oxygen concentrations, and too high starting dilution rates at the transition from fed-batch to chemostat mode (van Kessel et al., 2015; Camejo et al., 2017; Koch et al., 2019). Additionally, metagenomic studies have found urea uptake and metabolism genes frequent in comammox bacteria (Camejo et al., 2017; Palomo et al., 2018), and three urea-decomposing comammox enrichments were recently reported (Li et al., 2021), suggesting widespread capacity for urea utilization among comammox bacteria. Ammonia concentrations in our soils were low in April 2018, as well as in December 2017 (Huang et al., 2021), which could suggest that comammox bacteria in these soils may have adapted to primarily rely on urea and not on ammonia for growth, and that maybe other ammonia oxidizers in the soil may be less competitive for urea. Notably, Sakoula et al., 2020 found that *Ca.* Nitrospira kreftii was partially inhibited at total ammonium concentrations as low as 50 μM, further suggesting a potential importance of urea utilization. Such an ammonia-sensitive strain would have been strongly selected against in our batch enrichments. Further research is needed to elucidate the ecophysiology of comammox bacteria in EAA soils.

To our surprise, all three nutrient-limited reactors yielded a *Nitrosocosmicus* sp. as predominant ammonia oxidizer, accompanied by 2–3 different *Nitrospira* sp.-affiliated canonical nitrite oxidizers, suggesting that at the given temperature, pH, and media composition, this *Nitrosocosmicus* ecotype was the most competitive ammonia oxidizer under nutrient-limited chemostat conditions. Previous studies have suggested a pH and temperature-based niche separation of different soil AOA lineages based on *amoA* gene phylogeny and distribution (Gubry-Rangin et al., 2011, 2015; Prosser and Nicol, 2012). The observation that the same *Nitrosocosmicus* ecotype was enriched directly from soil and from a batch culture pre-enrichment, may corroborate this hypothesis, as all reactors in our study were maintained under the same temperature and pH regime. Our study joins a notably growing number of enrichment strategies yielding *Nitrosocosmicus* strains (Jung et al., 2016; Lehtovirta-Morley et al., 2016; Sauder et al., 2017; Liu et al., 2019). However, our results are particularly surprising since *Nitrosocosmicus* strains have thus far been noticed mainly for their observed higher tolerance to elevated ammonia concentrations compared to other *Nitrososphaeraceae*- and *Nitrosopumilaceae*-affiliated AOA strains (Lehtovirta-Morley et al., 2016; Sauder et al., 2017). Despite the relatively high ammonia tolerance, the highest growth rates of *Nitrosocosmicus* strains have thus far been reported at lower ammonia concentrations of 0.5 and 2 mM substrate concentrations (Jung et al., 2016; Lehtovirta-Morley et al., 2016; Sauder et al., 2017). Our results clearly indicate that at least some *Nitrosocosmicus* ecotypes also strongly compete under constantly limiting nutrient concentrations at or below 1 μM total ammonium. Thus far no kinetic data have been reported for *Nitrosocosmicus* strains and further physiological studies will be necessary to better understand the metabolism and kinetics of *Nitrosocosmicus* strains. Notably, neither the NS-δ-affiliated AOA strain detected in the batch culture, nor the NS-α-affiliated AOA strain detected in reactor B (Day 72) were further enriched (Figures 2, 3A,B), which may either confirm that these AOA may not be very competitive under nutrient limitation (Kits et al., 2017), or suggest that they may rely on organic carbon sources, antioxidants, or other components lacking in our growth media, or that their general growth conditions were not met well in our experiments.

After pH fluctuations in reactors A, B, and C we observed a small *Nitrosomonas* sp. population to regain in abundance, but then was outcompeted again by the *Nitrosocosmicus* after stabilization of pH (reactor A) and sub-cultivation (reactor B and C). This *Nitrosomonas* strain also highly dominated the original batch enrichment culture at 300 μM starting ammonium concentrations. Assuming that AOA and AOB both use ammonia as their primary substrate (Suzuki et al., 1974; Li et al., 2018), these observations may suggest that the *Nitrosocosmicus* strain exhibited only a marginally higher ammonia affinity compared to the *Nitrosomonas* strain. The rise in NH_3_ due to pH-dependent shifts of NH_3_ + H^+^ ⇔ NH_4_^+^ equilibrium may have been sufficient for the AOB strain to become more competitive in the chemostats and strongly dominate the batch culture at ammonium concentrations of 300 μM. Alternatively, the pH fluctuation could have impaired activity of the *Nitrosocosmicus* strain and may have caused slightly rising overall NH_4_^+^ concentrations in the reactors. It should be noted that based on the presented data we cannot determine how much the *Nitrosocosmicus* and *Nitrosomonas* strains each contributed to the overall rate of ammonia oxidation observed in the reactors. Further detailed kinetic studies on both strains and further enrichments targeting a wider range of temperature and pH regimes will be required to examine whether the niche separation hypothesis can be further substantiated with physiological evidence, or whether other cultivation-related biases were responsible for the observed outcome in our reactors.

Analysis of 16S rRNA gene amplicons revealed that a relatively large diversity of heterotrophic bacterial taxa persisted in the chemostat enrichments, suggesting that sufficient organic carbon was available to maintain this diversity of heterotrophs. Similar patterns of diverse heterotrophs maintained in nitrifier enrichments were previously observed in comammox enrichments (Takahashi et al., 2020). In our study enrichments with higher than 50% relative frequency of nitrifiers based on 16S rRNA gene amplicon sequences were obtained only for the *Nitrosomonas* strain in batch culture, at one time point in the original reactors, and more consistently only after additional sub-cultures established in reactors with elevated feed ammonia concentrations of 1.8 mM (Figure 2). Indeed, assuming a molar ratio of N oxidation to C fixation of approximately 5 for AOA and 8–12 for AOB, respectively (Billen, 1976; Prosser, 1989; Könneke et al., 2014), it can be estimated that using feed substrate concentrations of 300 μM N, ammonia oxidizers could build up between ∼ 60 μM (AOA) and 35 μM (AOB) biomass carbon under steady-state operation in reactors A–C. For comparison, organic carbon available from the 7.5 μM Fe EDTA [Fe C_10_H_16_N_2_O_8_] alone provided approximately 75 μM organic carbon source C. Given a carbon assimilation rate by heterotrophs of approximately 50% (Simon and Azam, 1989), the amount of EDTA supplied with the growth media was sufficient to build up approximately 35 μM biomass carbon. Thus, the biomass yield from organic carbon oxidation and nitrification could have been approximately similar during operation of reactors A–C, therefore explaining the relatively low enrichment factor of nitrifiers in our initial reactors. Interestingly, a notably higher enrichment of the *Nitrosomonas* strain was obtained in batch enrichment culture 9 with only 15.5% accompanying heterotrophs (Figure 2). Since we used the exact same media and ammonium feed concentration for reactor B, the frequent transfers and constant presence of nitrite may have limited the number of heterotrophic bacteria over time.

For the purpose of enrichment of nitrifiers long operation times, such as used for reactors A–C, may be detrimental, as in the late stages of the reactor operation the relative enrichment of nitrifiers actually declined (Figure 2). This decline may be due to slow accumulation of cell debris or cell aggregates that further fuel heterotrophic growth. Taken together, these observations suggest that 2–3 months reactor operation appears sufficient to obtain initial stable enrichments based on nutrient limitation alone and that further increases in ammonium feed is needed to obtain highly enriched nitrifier cultures. Furthermore, highly purified water and inorganic media sources, or reactor setups with biomass retention, are beneficial when operating reactors at low ammonium feed concentrations. Similar challenges will be associated with enrichment of potential mixotrophic nitrifiers that may rely on amendment of organic carbon or nitrogen compounds. Such organisms are predicted for example among AOA by observation of assimilation of amino acids (Ouverney and Fuhrman, 2000; Dekas et al., 2019) and isotopic signals of organic carbon assimilation in archaeal membrane lipids (Ingalls et al., 2006).

Treatment of the enrichments with antibiotics or combination of antibiotics and organic carbon sources or antioxidants did not result in pure *Nitrosocosmicus* cultures, instead either ammonia oxidation ceased completely, or if activity was present, the antibiotic treatment did not suppress growth of bacteria. Although it seems unlikely that the *Nitrosocosmicus* strain in our study was directly inhibited by antibiotics since the same antibiotics have previously been used successfully for other *Nitrosocosmicus* enrichments (Lehtovirta-Morley et al., 2016; Liu et al., 2019), we cannot completely rule out cytotoxic effects of the antibiotics on this strain, especially because it was selected for only based on nutrient limitation and in the complete absence of antibiotics. Nonetheless, more likely our results suggest an indirect inhibition via suppression of heterotrophic bacteria, that provide a thus far unknown benefit to the *Nitrosocosmicus* strain. Notably, a similar inhibition of activity in the presence of antibiotics was also observed in *Ca.* Nitrosocosmicus hydrocola G61 (Sauder and Neufeld, personal communication, Sauder et al., 2017). And neither in G61, nor in our enrichment this inhibition could be alleviated by addition of organic carbon compounds shown to stimulate activity of strain G61 (Sauder et al., 2017). Additional experiments will be required to test if supplementation of other potential carbon sources including amino acids, sugars, and complex substrates (peptone, yeast extract, and casamino acids), shown to stimulate activity of other *Nitrosocosmicus* strains (Jung et al., 2016; Sauder et al., 2017) can alleviate this inhibition, or whether more complex kinds of interactions between the *Nitrosocosmicus* strain and heterotrophic bacteria may be responsible for the observed dependence of the *Nitrosocosmicus* strain on heterotrophic bacteria.

## Data Availability Statement

DNA sequences obtained in this study have been deposited at NCBI Sequencing Read Archive under BioProject # PRJNA728812 (16S rRNA gene amplicon sequences) and GenBank accession numbers MZ196446-MZ196452 (amoA gene sequences).

## Author Contributions

SC and WM-H designed the research. SC, JR, EC, NS, SS-M, and WM-H conducted enrichments and bioreactor experiments. JR and WM-H wrote the manuscript. All authors conducted molecular analyses.

## Conflict of Interest

The authors declare that the research was conducted in the absence of any commercial or financial relationships that could be construed as a potential conflict of interest.
