# Supplementary material for: Nutrient-Limited Enrichments of Nitrifiers From Soil Yield Consortia of Nitrosocosmicus-Affiliated AOA and Nitrospira-Affiliated NOB
Source: Front Microbiol. 2021 Jul 12;12:671480. doi: 10.3389/fmicb.2021.671480 (PMC8312096; doi:10.3389/fmicb.2021.671480)
Supplement: Supplementary file 1 [file Data_Sheet_1.pdf]

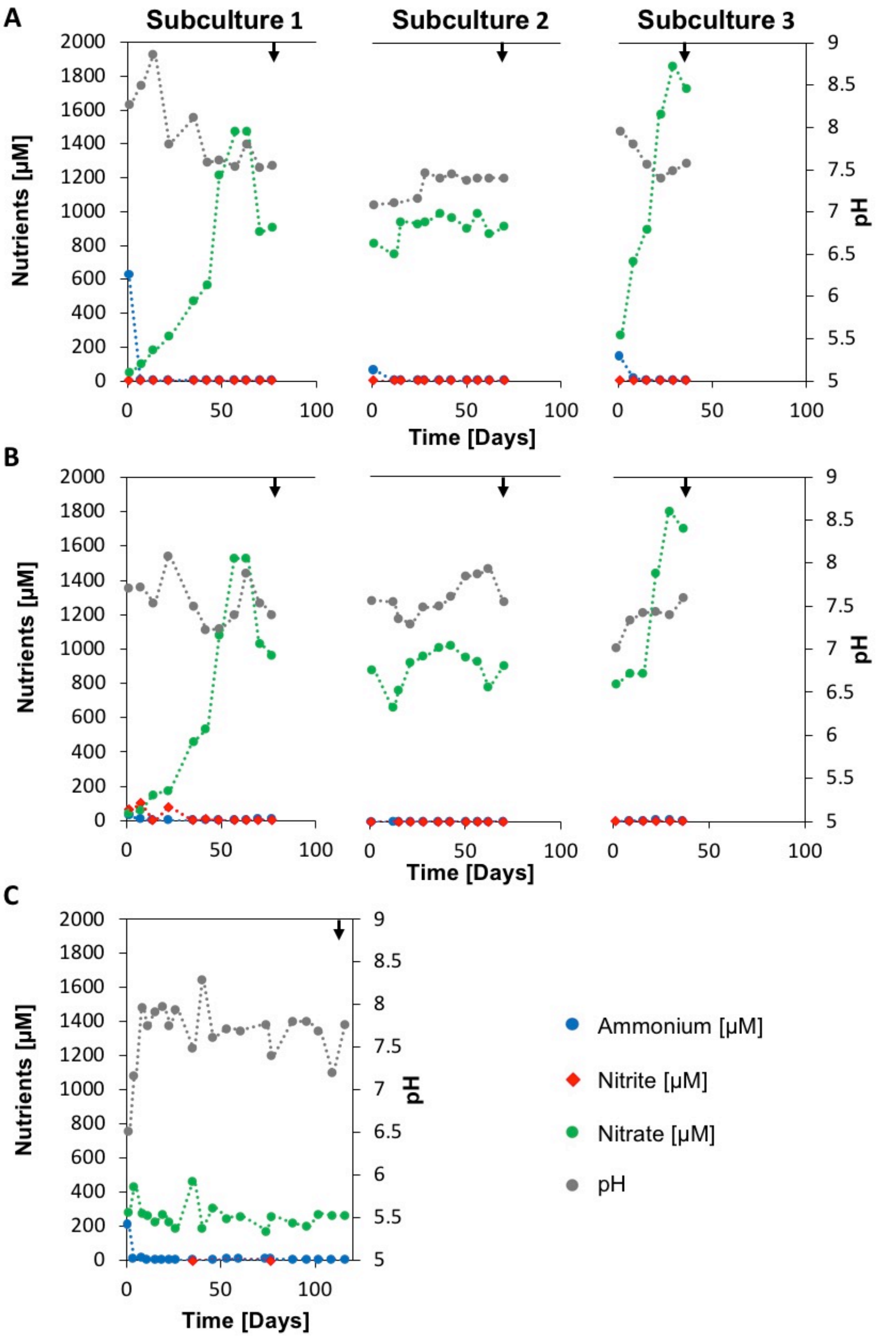

Supplementary Figure 1. Time course of nutrients and pH in subculture chemostat reactors inoculated with samples from reactors A, B, and C, respectively. Subculture 1, 2, and 3 refer to sequential subcultures of reactors shown in Figure 1. Arrows indicate sampling points for amplicon sequencing analysis. All subculture 1 reactors were seeded with 100 ml culture liquid of reactors A, B, and C, respectively, following the last DNA sampling time point (arrows in Figure 1). Subculture 2 reactors were seeded with inoculums (~800 ml) from subculture 1 reactors after DNA samples had been taken, and subculture 3 reactors were seeded with sample from subculture 2 reactors and feed ammonium concentration was raised to 1.5 mM. Subculture A1 and B1 were accidentally fed with 1.5 mM ammonium feed between Day 54 and 65.
